# Modeling and Sensitivity Analysis of the Forward Osmosis Process to Predict Membrane Flux Using a Novel Combination of Neural Network and Response Surface Methodology Techniques

**DOI:** 10.3390/membranes11010070

**Published:** 2021-01-19

**Authors:** Jasir Jawad, Alaa H. Hawari, Syed Javaid Zaidi

**Affiliations:** 1 Centre for Advanced Materials, Qatar University, P.O. Box 2713, Doha, Qatar; jasir.jawad@qu.edu.qa (J.J.); szaidi@qu.edu.qa (S.J.Z.); 2 Department of Civil and Architectural Engineering, Qatar University, P.O. Box 2713, Doha, Qatar

**Keywords:** artificial neural network, forward osmosis, water treatment, desalination, response surface methodology

## Abstract

The forward osmosis (FO) process is an emerging technology that has been considered as an alternative to desalination due to its low energy consumption and less severe reversible fouling. Artificial neural networks (ANNs) and response surface methodology (RSM) have become popular for the modeling and optimization of membrane processes. RSM requires the data on a specific experimental design whereas ANN does not. In this work, a combined ANN-RSM approach is presented to predict and optimize the membrane flux for the FO process. The ANN model, developed based on an experimental study, is used to predict the membrane flux for the experimental design in order to create the RSM model for optimization. A Box–Behnken design (BBD) is used to develop a response surface design where the ANN model evaluates the responses. The input variables were osmotic pressure difference, feed solution (FS) velocity, draw solution (DS) velocity, FS temperature, and DS temperature. The R2 obtained for the developed ANN and RSM model are 0.98036 and 0.9408, respectively. The weights of the ANN model and the response surface plots were used to optimize and study the influence of the operating conditions on the membrane flux.

## 1. Introduction

Forward osmosis is an osmotically driven process in which water molecules permeate through a semi-permeable membrane [1]. The driving force in forward osmosis (FO) is the osmotic gradient between a dilute feed solution and a concentrated draw solution. The most common transport models studied for the FO process are based on the film theory concept [2,3,4]. The development of these mathematical models requires extensive knowledge of the process as well as the evaluation of the membrane, solute, and solvent properties, which adds more complexity to the process [5,6].

The artificial neural network is a simple and efficient modeling technique based on the working of biological neurons. It is a data-driven technique that uses a learning algorithm to develop a relationship between input and output variables. Applications of artificial neural network (ANN) include function approximation, clustering, pattern recognition, and image processing. A few advantages of ANN include its ability to modeling complex non-linear systems; to work with incomplete, noisy or missing data; no effect of the data distribution and interactions among the factors; and continuous improvement of the model by updating the model with new data set [7].

The trend is shifting towards artificial intelligence-based modeling techniques such as ANN, as several studies have been published in literature over the last two decades. Niemi et al. reported a 50% decrease in the computational time for the neural network compared to the conventional ultrafiltration model [8]. Corbatón-Báguena et al. identified that the normalization of the data and randomization of weights improved the performance of the model [9]. Darwish et al. compared different training algorithms and found the Bayesian regularization training algorithm to be the fastest and more stable towards initial random weights and biases [10].

ANN has been used widely to predict membrane flux and study membrane fouling in ultrafiltration, nanofiltration and reverse osmosis [11,12,13,14,15]. Yang et al. predicted membrane flux and specific energy consumption of a vacuum membrane distillation based on operating conditions and membrane length using ANN model [16]. Khayet et al. developed response surface methodology (RSM) and ANN models based on experimental designs for the simulation and optimization of the reverse osmosis (RO) desalination pilot plant [15]. The models were compared by studying the effect of feed quality and operating conditions on the RO performance. Rahmanian and coworkers compared RSM, ANN, and adaptive neuro-fuzzy inference system (ANFIS) modeling techniques using a set of operating conditions to predict membrane flux and rejection rate for the removal of lead ions [13]. ANN models have found their application in optimization and control as well. Liu et al. modeled and optimized the microfiltration process with turbulence promoter to maximize Flux Improvement Efficiency (FIE) using ANNs [17]. Cabrera et al. developed an ANN model that predicted flow and pressure setpoint based on the varying power supplied from the wind turbine to RO desalination plant while keeping the recovery rate within a particular range to control the desalination process [18]. Neural networks have shown superiority over the conventional models so far. However, it does come with its own limitations. As Abbas and Al-Bastaki showed, ANNs can be reliable for interpolation, but extrapolation leads to inaccurate results [14].

Only two efforts have been made towards ANN modeling of the forward osmosis process. Pardeshi et al. developed a Taguchi-neural approach to determine the optimum operating conditions of the FO groundwater desalination to maximize the reserve solute flux selectivity [19]. The study also evaluated the importance of the parameters using analysis of variance (ANOVA) that could affect the quality characteristics of FO. In our previous study, an ANN model for a generalized prediction of membrane flux for lab-scale FO desalination [20]. The model was compared to multiple linear regression and published mathematical models, which showed satisfactory performance. However, the model was too complex to analyze the weights associated with the input variables due to multiple hidden layers having a high number of neurons. Table 1 summarizes some of the characteristics of ANN models and their performance developed for the membrane-based separation processes.

Optimization methods often take a considerable amount of computational time and cost for numerical simulation, whereas RSM reduces the computational cost and performs more efficiently [22]. Rahmanian et al. presented a study to maximize the lead removal from aqueous solution for micellar-enhanced ultrafiltration using response surface methodology [23]. The three-factor and three-level experimental design were based on the Box–Behnken design and a fuzzy logic model developed to predict membrane flux and metal rejection. Garg and Joshi used RSM to maximize the recovery rate and salt rejection and minimize energy consumption for the RO process while also studying the relative significance using centre composite design (CCD) [24]. Mansouri et al. coupled the RSM and computation fluid dynamics (CFD) model to investigate the mass transfer and hydrodynamics in a feed channel of spiral wound membrane [22]. The design points were generated using the Latin hypercube sampling method. The methodology helped identify the most and least effective parameters on the optimum conditions.

Several RSM studies on forward osmosis process have been published in the literature. Zaviska and Zou studied the potential of forward osmosis as a pre-treatment to RO using response surface based on CCD [25]. The experimental model was used to calculate the energy consumption using water flux, water recovery, and final DS concentration. Khayet et al. optimized a solar FO pilot plant with the application of statistical experimental design and RSM by studying the effect of operating conditions on FO performance index and energy consumption [26]. Minier-Matar et al. applied Box–Behnken design (BBD) and RSM techniques to study the optimization of the FO process for diluting concentrated brine from desalination plants by osmotically injecting water from Produced and process water from oil and gas operations [27]. Zhou et al. presented the modeling and optimization of the combined FO and membrane distillation (MD) process using BBD to generate the response surface points [28]. The flow rate and concentration of both feed solution (FS) and draw solution (DS) were used to maximize the membrane flux and removal efficiency. Naghdali et al. investigated a FO process with an aquaporins-based membrane and maximized chromium rejection and membrane flux using RSM [29]. FS concentration, DS concentration, and time were the critical parameters optimized in their study.

Literature has shown that although theoretical models are universal, these are not as reliable and accurate as of the machine learning models such as neural networks. So, there is a tradeoff between the universality and accuracy of the prediction. Furthermore, the ANN models have the advantage of training the model with newer data. Therefore, the predictions can be continuously improved. For this specific study, the experimental data were generated using only a single type of membrane. Therefore, its applicability is limited to this membrane. However, the methodology shown is general and a training model with different types of membrane data will allow for the consideration of specific membrane properties such as porosity, tortuosity etc.

To the best of our knowledge, the weights of the FO ANN model have not been analyzed to find the relative importance of the operating conditions on the membrane flux. In this work, a combined ANN-RSM approach was used to model and optimize the membrane flux based on five operating conditions, i.e., osmotic pressure difference, FS velocity, DS velocity, FS temperature, and DS temperature. The novel methodology presented in the paper provides an alternative for developing RSM model without performing experiments based on the experimental design. Instead, it uses ANN model to fill in the experimental design data for the RSM model. Initially, our published study on the combined influence of crossflow velocity and temperature on the FO process (AL-DS orientation) was used to model neural network. The weights of the ANN model were studied to calculate the relative importance of the input variables on the membrane flux to investigate the sensitivity of the model. The RSM technique requires an experimental design to work with, while ANN does not require one [15]. Therefore, a response surface design is generated with the help of BBD in which the responses are evaluated using the developed ANN model. The response surface plots were used to optimize flux and study the impact of the operating conditions. 

## 2. Materials and Methods

### 2.1. Experimental Design and Data Processing

In this work, data from our previous study by Hawari et al. has been used to develop the ANN model [30]. The experiments were conducted on SEPA CF forward osmosis cell supplied by Sterlitech™ Co, Kent, Washington, USA. Thin Film Composite (TFC) (Hydration Technology Innovations, Albany, Oregon, USA) was composed of polyester (PE) mesh embedded on polysulfone (PSF) substrate to form the support layer. The polyamide dense active layer of the membrane was formed by interfacial polymerization. The study investigated the combined influence of the temperature and flow rate on the performance of the FO process. The active layer of the membrane faced the draw solution (AL-DS or PRO mode), as this orientation resulted in higher membrane flux. This is because, for AL-DS orientation, dilutive external concentration polarization takes place on the DS side, whereas concentrative internal concentration polarization (ICP) takes places at the FS side [31]. The effect of concentrative ICP is much less severe than dilutive ICP; therefore, the flux is higher in AL-DS than in AL-FS. Each experiment was run for 30 min to calculate the average membrane flux. The feed solution was either distilled water or 0.086 M NaCl solution. The draw solution was a 0.5 M NaCl solution. The experiments were run in a batch mode with no recycling of the feed or draw solutions. The membrane flux was calculated by measuring the change in the weight of the feed solution using Equation (1).

(1) $$J_{w}=\frac{\left(W_{t}-W_{0}\right)}{A_{m}\Delta t}$$

where $J_{w}$ is the membrane flux, $W_{0}$ and $W_{t}$ are the initial weight and weight at time t of the feed solution, $A_{m}$ is the membrane area, and Δt is the time interval. The van’t Hoff equation, given in Equation (2), was used to calculate the osmotic pressure of the feed and the draw solution. The osmotic pressure difference is given by Equation (3)

(2) π=iRTC

(3) $$\Delta \pi =\pi_{DS}-\;\pi_{FS}$$

where i is the van’t Hoff coefficient, R is the universal gas constant, T is the temperature, C is the molar concentration of the solution, and $\pi_{DS}$ and $\pi_{FS}$ are the osmotic pressure of the draw and the feed solution, respectively. 

The description of the input and output variables for the neural network model is given in Table 2. The selection of these 5 input variables was due to the reason that only these variables were varied in the previous study. Parameters like pH, membrane type and orientation were kept constant for the experiments conducted in the study. The FS and DS composition was incorporated in the form of osmotic pressure difference as this is the driving force for the separation in FO. A total of 76 data points were extracted from the study on which the ANN model was trained and tested (refer to Supplementary data).

The experimental data is divided into three sets, i.e., training, validation, and test dataset with a ratio of 80%, 10%, and 10%, respectively. The data was non-randomized for the sake of consistency and comparison between different developed networks for the selection of the best model. The data is normalized to avoid numerical overfitting due to very large or very small weights associated with the data before the training of the ANN model [32,33]. In this study, the data, including both inputs and outputs, were normalized in the range of −1 to 1 using the MATLAB’s ‘mapminmax’ function given by the Equation (4).

(4) $$y=\;\frac{2\left(x-x_{\min}\right)}{\left(x_{\max}-x_{\min}\right)}+1$$

where y is the normalized value of x, and $x_{\max}$ and $x_{\min}$ are the maximum and minimum value of the data, respectively.

### 2.2. Artificial Neural Network Model

**Figure 1:**
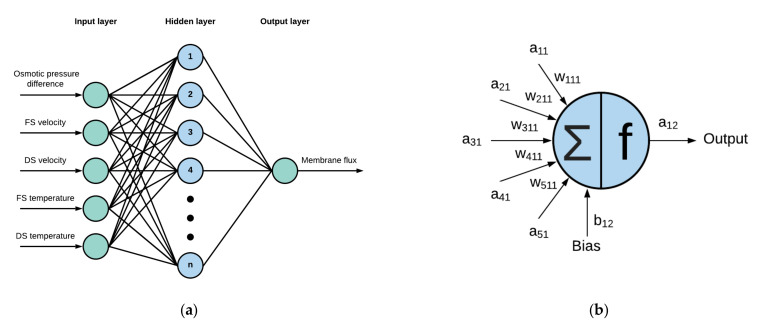
(**a**) The network architecture of multi-layer perceptron neural network model consisting of a single hidden layer, five inputs, and a single output, (**b**) Close-up of the hidden neuron representing the evaluation of neuron’s output value.

In this work, a multi-layer perceptron (MLP) was developed to model the FO process to predict the membrane flux. Figure 1a shows an MLP-ANN model’s network architecture with five input neurons and a single output used in this study. An MLP type neural network consists of input, hidden, and an output layer of neurons. There can be multiple hidden layers within the ANN. However, a multi-layer feedforward network with a single hidden layer is considered to be a universal approximator [34]. Adding multiple layers also adds complexity in the model with increased computational time and effort. A new hidden layer shall be added in cases where the performance of the ANN is insufficient even at high number of neurons in the single hidden layer. Analysis of the weights and its physical significance is also possible with single hidden layered network, whereas it will become too complex for multi-layered (hidden) model.

In general, the ANN modeling consists of three phases for the three different datasets, i.e., training, validation, and test phase. During the training phase, initially, the weights are assigned to the neurons using a training algorithm or optimization method. For this study, the weights and biases were initialized using the Nguyen–Widrow initialization method [35]. Furthermore, the Levenberg–Marquardt (LM) algorithm was used to train the network and update the weights due to its high performance and convergence speed [36,37,38]. The output of each neuron is related to the neurons and weights of the previous layer except for the input layer, as given in the Figure 1b. The value of the neuron output is evaluated using Equation (5).

(5) $$a_{ij}=f_{j}\left\{\overset{n_{\left(j-1\right)}}{\underset{k=1}{\sum }}\left(\;a_{k\left(j-1\right)}w_{ki\left(j-1\right)}\right)+\;b_{ij}\right\}$$

where $a_{ij}$ and $b_{ij}$ are the output and bias of the *i*th neuron in the *j*th layer, $a_{k\left(j-1\right)}$ and $w_{ki\left(j-1\right)}$ are the output and the weight of neuron from the previous layer respectively, $n_{\left(j-1\right)}$ is the neurons in the hidden layer $\left(j-1\right)$, and $f_{j}$ is the activation or transfer function of the *j*th layer.

The activation or transfer function adds non-linearity to the neural network [39]. The most commonly used activation functions are logistic sigmoid (log-sigmoid), hyperbolic tangent sigmoid (tan-sigmoid), and pure linear functions (purelin). The log-sigmoid function has an output range from 0 to 1, whereas it is −1 to 1 for the tan-sigmoid function. In this study, the output layer was set to purelin function (−∞ to ∞), while the hidden layer switched between log-sigmoid and tan-sigmoid activation function in order to find the best network.

During the learning phase, the weights are constantly updated by minimizing the error between the targeted and predicted output variable. This process is called supervised learning and goes on until the required criterion is met, i.e., minimum error, number of validation checks, etc. The performance of the trained network can be calculated using several indicators given by Equations (6)–(10).

(6) $$\mathrm{Sum}\;\mathrm{square}\;\mathrm{error}\;\left(SSE\right)=\sum_{i=1}^{N}{\left(y_{p,i}-y_{t,i}\right)}^{2}$$

(7) $$\mathrm{Mean}\;\mathrm{square}\;\mathrm{error}\;\left(MSE\right)=\frac{1}{N}\sum_{i=1}^{N}{\left(y_{p,i}-y_{t,i}\right)}^{2}$$

(8) $$\mathrm{Root}\;\mathrm{mean}\;\mathrm{square}\;\mathrm{error}\;\left(RMSE\right)=\sqrt{\frac{1}{N}\sum_{i=1}^{N}{\left(y_{p,i}-y_{t,i}\right)}^{2}}$$

(9) $$\mathrm{Determination}\;\mathrm{coefficient}\;(R^{2})=1-\;\frac{\sum_{i=1}^{N}\left(y_{p,i}-y_{t,i}\right)}{\sum_{i=1}^{N}\left(y_{p,i}-y_{m}\right)}$$

(10) $$\mathrm{Adjusted}\;R^{2}=1-\;\frac{\left(1-R^{2}\right)\left(N-1\right)}{N-k-1}$$

where $y_{p,i}$ and $y_{t,i}$ are the predicted and target values of membrane flux, respectively; N is the total number of data points; $y_{m}$ is the mean of the actual value of water; k is the total number of input variables.

The validation phase of the ANN modeling helps in avoiding the over-training of the network. If the trained network continues to perform well, but at the expense of decreasing performance when subjected to the validation dataset, it means that the model is over-fitted and may not be able to generalize well. Lastly, the test phase of the ANN modeling begins where the final trained network is subjected to unseen data to check its generalized prediction capability. The ANN modeling was implemented using MATLAB and Neural Network Toolbox 11.1, The MathWorks, Inc., Natick, Massachusetts, United States.

The connection weights of the neural network represent the link between the input (cause) and output (effect). Therefore, the relative importance (RI) of each input variable is calculated using the weight partitioning methodology (Equation (11)) to demonstrate the sensitivity of the ANN model [40,41].

(11) $$RI_{m}\%=\;\frac{\sum_{j=1}^{n_{h}}\left\{\left(\frac{i_{mj}}{\sum_{k=1}^{n_{m}}i_{kj}}\right)O_{j}\right\}}{\sum_{l=1}^{n_{m}}\left[\sum_{j=1}^{n_{h}}\left\{\left(\frac{i_{mj}}{\sum_{k=1}^{n_{m}}i_{kj}}\right)O_{j}\right\}\right]}\;\times 100$$

where m is the number of input neurons, $n_{h}$ the number of hidden neurons, $i_{mj}$ is the absolute value of connection weights between the input m and hidden neuron j, and $O_{j}$ is the absolute value of connection weights between the hidden neuron j and the output.

### 2.3. Response Surface Methodology

Response surface methodology (RSM) is an optimization technique that is used to determine the combination of input factors that minimizes or maximizes the objective function [15]. Combined with design of experiments (DoE), RSM second-order polynomial regression models can predict the performance of any system. Furthermore, it can be used to determine the relative significance of multiple factors in the presence of complex interactions between them using minimal experimental design [26]. In this study, Box-Behnken Design (BBD) was implemented to generate the design to investigate the performance of the FO process. The advantage of the BBD method is that it does not include the combinations where all factors are either at their highest or at their lowest simultaneously [23]. This is useful to avoid the extreme conditions in the design. The developed ANN model is used to evaluate the membrane flux for the 5 factors and 3 level design resulting from BBD. Therefore, the discrete design space from ANN is converted to a continuous design space using RSM. The BBD generated a set of 46 data points using the lower and upper levels for each input variable are given in Table 2. The general coded form of the second-order polynomial equation resulting from the response surface regression is given by Equation (12) [42].

(12) $$\hat{Y}=\beta_{0}+\overset{n}{\underset{i=1}{\sum }}\beta_{i}X_{i}+\overset{n}{\underset{i=1}{\sum }}\beta_{ii}X_{i}^{2}+\overset{n}{\underset{i<j}{\sum }}\beta_{ij}X_{i}X_{j}$$

where $\hat{Y}$ is the predicted variable, $X_{i}$ denotes the input variable, $\beta_{0},\beta_{i},\beta_{ii},\;\beta_{ij}$ refers to the regression coefficients, and n is the number of input variables. Minitab^®^ (State College, PA, USA) was used to create the Box–Behnken design and the RSM. The response surface methodology was implemented using Minitab^®^.

## 3. Results

### 3.1. Neural Network Model Selection and Performance

**Figure 2:**
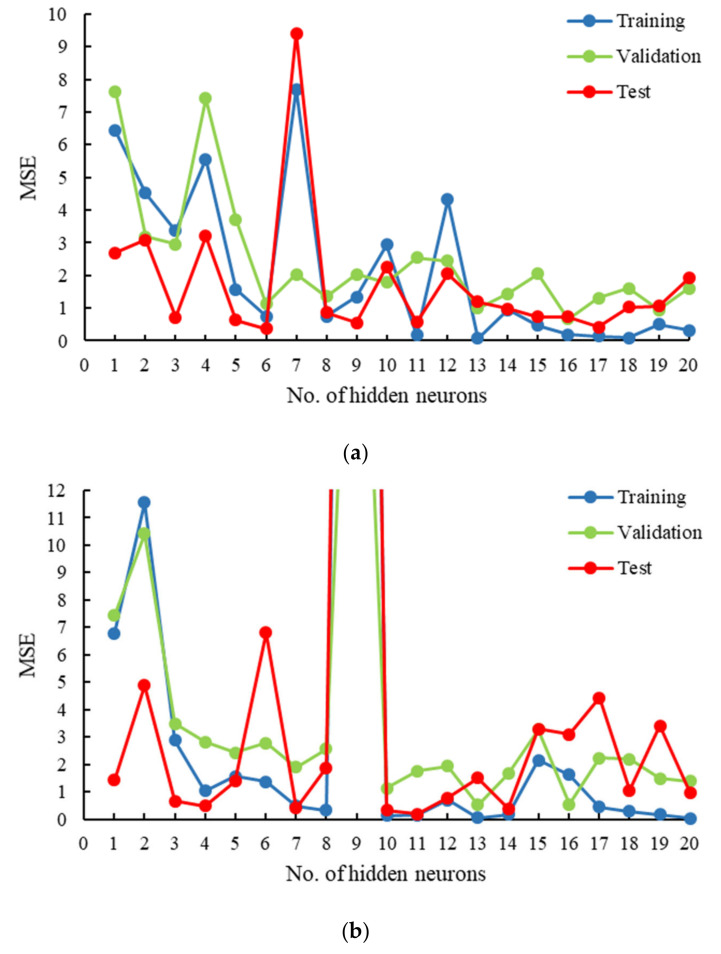
Simulation summary for selecting the optimum network using mean squared error (MSE) at different number of hidden neurons and activation function as (**a**) log-sigmoid, and (**b**) tan-sigmoid.

Several networks were trained to model the FO process using osmotic pressure difference between the feed and draw solution, flow velocity of the feed and draw solution, and temperature of the feed and the draw solution as input parameters, whereas membrane flux as an output parameter. Single hidden layered networks with either log-sigmoid or tan-sigmoid as activation functions having neurons ranging from 1–20 were trained separately. For this study, the performances of the networks are compared using mean squared error (MSE). The selection criteria for choosing the optimum network was based on selecting the model with the least training error without compromising its efficiency in the validation and testing phases. Figure 2a shows the MSE in all three phases of trained models at hidden neurons from 1 to 20 and log-sigmoid as the activation function. The highest MSE was observed for the model with 7 hidden neurons, whereas the error was relatively low for hidden neurons 13 or more. Similarly, Figure 2b shows the MSE in all three phases of trained models at hidden neurons from 1–20 and tan-sigmoid as the activation function. An abrupt increase in MSE was observed for the model with 9 hidden neurons in each phase. It is not possible to determine the exact cause of high MSE at specific number of neurons in the hidden layer. The MSE does not follow a specific trend with respect to the increasing number of neurons in the hidden layer. However, in general, MSE is high at very low number of neurons and it improves as number of neurons or hidden layers are added to the network but to a specific limit. The limiting factor is sometimes the over-training of the network, which may again lead to high MSE. Due to this reason, a range of 1–20 neurons is visualized in order to determine the best possible model (lowest MSE) in the training phase without comprising its efficiency in validation and testing phases. The model with 10 hidden neurons showed one of the least training phase MSE, as well as high performance in the validation and testing phase. This criterion allowed for the development of a high-performance network as well as a model with generalization capabilities. 

**Figure 3:**
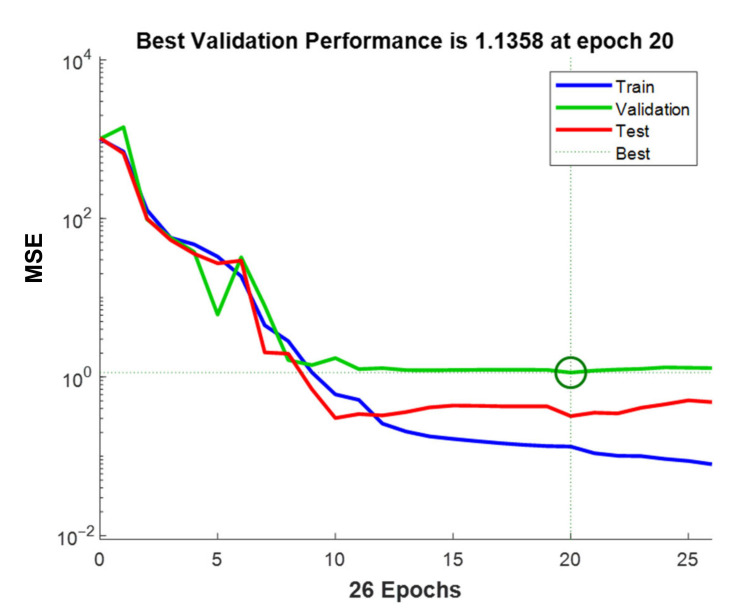
Mean squared error (MSE) evolution of all phases for the optimum network.

The network topology for the optimum model was 5-10-1 (Input-Hidden Neuron-Output). The evaluation of MSE during the training process can be visualized for all three datasets in Figure 3. An epoch marks the updating of weights during the training phase. At 20 epochs, the best validation performance was reached and, therefore, accepted as the best performance for this network architecture. The continuous training beyond 20 epochs showed a decrease in training phase MSE. However, the validation and testing phase MSE was increasing. This means that the network was over-training beyond 20 epochs resulting in a low generalization efficiency. It was also interesting to see that the MSE was higher for the validation phase than the test phase, which did not participate in the training process. However, it is purely coincidental that the MSE is higher for validation and less for the test phase. The vice versa can be seen in Figure 2b (MSE higher for test phase and lower for validation phase) for the case of 6, 13, 16, 17, and 19 number of neurons in the hidden layer. Possible reasons for this phenomenon may include the type of data used in each phase that is training, validation and testing.

Table 3 shows the MSE, RMSE, SSE, and R^2^ for the individual and overall dataset of the optimum model. The R-value, correlation coefficient, is obtained from the MATLAB code that can be squared to calculate the coefficient of determination (R^2^), which is a tool to determine the ‘goodness of fit’ between the target and predicted variables. The R^2^ of training, validation, test, and the overall dataset was 0.99906, 0.99295, 0.99776, and 0.98036, respectively, where a value of 1 means a perfect fit. This means that about 99% of the variability in each phase between target and predicted variable can be explained by the ANN model. 

**Figure 4:**
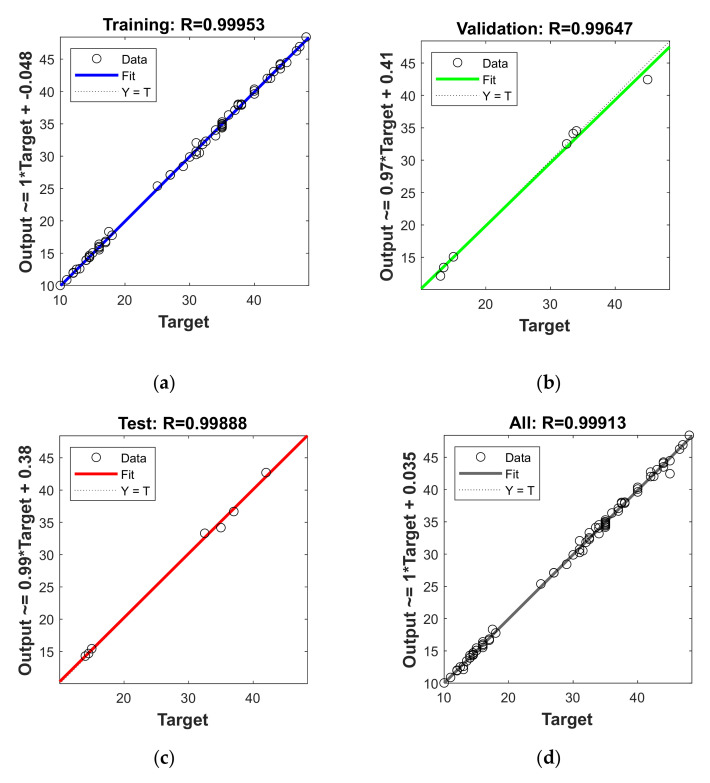
Comparison between the targeted and predicted output values in (**a**) training phase, (**b**) validation phase, (**c**) test phase, (**d**) overall dataset, of the artificial neural network (ANN) model.

Figure 4 shows the regression analysis with its equation in each phase between target and predicted output. The training and validation dataset simulations, presented in Figure 4a,b, respectively, are involved in the training of the ANN model. The correlation coefficient is 0.99953 for training and 0.99647 for the validation phase. The two phases consisted of data points distributed over the range of the input variables to minimize the extrapolation as much as possible. This is because the ANNs have proven to be a useful tool for interpolation. However, extrapolation may lead to inaccurate results [14,37]. The test phase presented in Figure 4c shows excellent agreement between the predicted and targeted output, despite being unseen data subjected to model. The R-value, in this case, was found to be 0.99888. Figure 4d presents regression over the predicted and targeted variables of the complete dataset, including all three datasets with a high correlation coefficient of 0.99913.

### 3.2. Neural Network Sensitivity Analysis

In our previous study, an ANN model was developed with nine input variables to predict the membrane flux, but the weights associated with the network were not analyzed for their contribution towards the output [20]. In this paper, the relative importance of each input variable was assessed on the ANN model output (membrane flux) using a sensitivity analysis approach that employs the partitioning of the weights. The weights and biases for the optimal ANN model are given in Table 4. It is to be noted that these weights are for the normalized input and output. 

**Figure 5:**
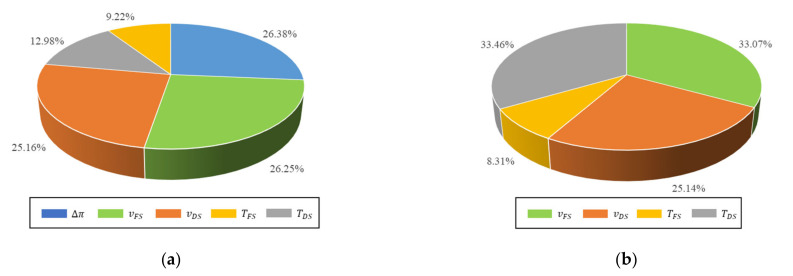
The relative importance of the FO parameters (**a**) On membrane flux based on ANN weights of the optimal model in this study, (**b**) On membrane flux to reverse salt flux ratio based on analysis of variance (ANOVA) from a published study by Pardeshi et al. [19].

The relative importance percentage for the ANN model inputs was calculated using Equation (11) and presented in Figure 5a. The order of importance was determined to be osmotic pressure difference > FS velocity > DS velocity > DS temperature > FS temperature. According to the calculations, it is noted that the relative importance of the osmotic pressure difference, FS velocity, and DS velocity is comparable. The FS temperature has the least effect on the membrane flux, whereas osmotic pressure difference has the highest impact. Pardeshi et al. analyzed the percentage contribution of each input on the ratio of membrane flux to reverse salt flux using F-test value from ANOVA [19]. The study revealed the contribution order of DS temperature > FS velocity > DS velocity > FS temperature presented in Figure 5b. The osmotic pressure difference was not included in their study. The impact of both velocities and FS temperature was found to be comparable. However, the DS temperature showed the highest impact on the reverse solute flux selectivity, contrary to the analysis in this study. The difference in the sensitivity analysis can be the result of the dispersion of the data [43]. The dispersion of the data may induce a different impact of the input variables on the response variable.

### 3.3. Application of Response Surface Methodology

In this work, BBD was used to investigate the optimal conditions of the FO process by building response surface plots. The range of these input factors was the same as given in Table 2. The design parameters and corresponding responses are shown in Table 5. The 25% increase in osmotic pressure leads to an increase in the value of flux by one order of magnitude (std. order 1 and 2 in Table 5). This is also true because osmotic pressure is the driving force for the separation in the forward osmosis process. It also corresponds to one of the findings of the work, which is that the osmotic pressure has the highest impact on the value of the flux. The model shows high predictive efficiency but still comes with a small error that may be translated to a much bigger error if extrapolating. The response (membrane flux) was calculated using the optimal ANN model at the BBD specified conditions. The implementation of RSM resulted in a second-order polynomial regression equation whose uncoded form is written as:

(13) $$\begin{array}{ll} J_{w}=905-86.2\Delta \pi & +0.97v_{FS}-4.36v_{DS}+4.60T_{FS}-2.20T_{DS}+1.831\Delta \pi^{2}-0.0179v_{FS}^{2}+0.0299v_{DS}^{2}\\ & -0.0272T_{FS}^{2}+0.0414T_{DS}^{2}-0.0190\Delta \pi v_{FS}+0.2859\Delta \pi v_{DS}+0.037\Delta \pi T_{FS}+0.157\Delta \pi T_{DS}\\ & +\;0.0264\;v_{FS}T_{FS}-\;0.0778\;v_{FS}T_{FS}+\;0.0867v_{FS}T_{DS}+\;0.0076\;v_{DS}T_{FS}-\;0.1467v_{DS}T_{DS}\\ & -\;0.0875\;T_{FS}T_{DS} \end{array}$$

The significance of the response surface model was statistically analyzed using ANOVA, and the results are presented in Table 6. The model F-value obtained was 19.85, where the *p*-value was smaller than 0.001. Moreover, the R^2^ value of 0.9408 and the adjusted R^2^ of 0.8934 was obtained. This means that 94.08% deviations can be explained by the RSM model, and the high value of adjusted R^2^ showed that input factors considered in this study were significant. All these indicators showed that the RSM model is statistically acceptable.

**Figure 6:**
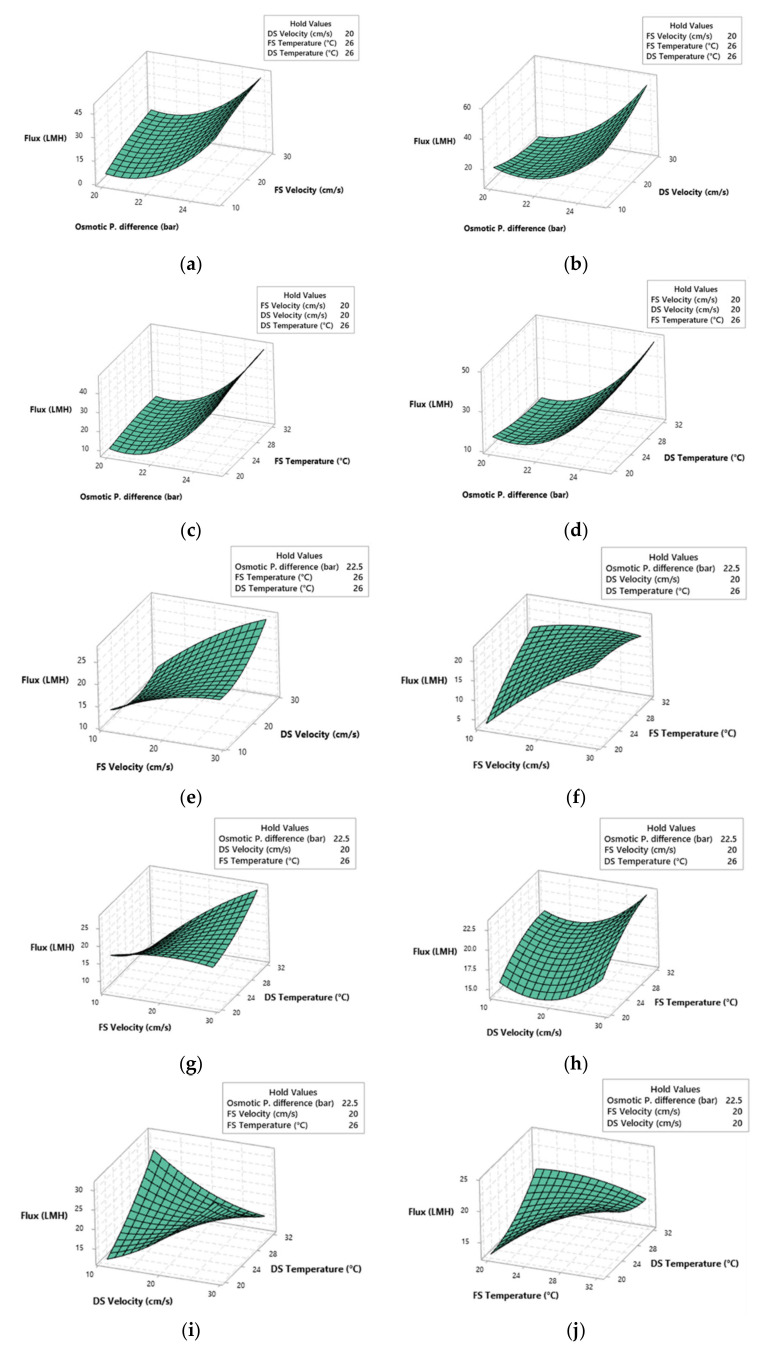
Response surface plot of the predicted membrane flux $J_{w}$ as a function of (**a**) Δπ and $v_{FS}$, (**b**) Δπ and $v_{DS}$, (**c**) Δπ and $T_{FS}$, (**d**) Δπ and $T_{DS}$, (**e**) $v_{FS}$ and $v_{DS}$, (**f**) $v_{FS}$ and $T_{FS}$, (**g**) $v_{FS}$ and $T_{DS}$, (**h**) $v_{DS}$ and $T_{FS}$, (**i**) $v_{DS}$ and $T_{DS}$, (**j**) $T_{FS}$ and $T_{DS}$.

Figure 6 shows the response surface plots for the prediction of membrane flux using the five input variables. For each plot, three of the input values were held constant (range average) while variations occur in the other two. Figure 6a–d shows the impact of osmotic pressure difference and the other input variables on the membrane flux. In all the cases, the increase in the osmotic pressure difference showed a positive influence on the membrane flux. This is true as the driving force for the FO process is the osmotic pressure difference. Moreover, the highest membrane flux (up to 55 LMH) was obtained at the maximum osmotic pressure difference of 25 bar and the DS velocity of 29 cm/s, as shown in Figure 6b. Similarly, the increase in FS velocity also showed a positive impact on the flux, as seen in Figure 6a,e–g. The impact of FS velocity was more significant at low FS temperature than at high FS temperature and vice versa for the DS temperature. A comparison between the variation of both FS and DS velocities in Figure 6e showed that the simultaneous increase in both velocities would result in a high membrane flux. At DS velocity of 11.0 and 29.0 cm/s, the change FS velocity seemed to have a proportional response towards the membrane flux. It was interesting to note that, at a fixed FS velocity of 11.0 cm/s, increasing DS velocity first resulted in a decrease and then an increase in the membrane flux. While at a fixed FS velocity of 29.0 cm/s, the increase in DS velocity results in an increase in the membrane flux. This could mean that the prediction of the membrane flux is more sensitive towards the FS velocity. The DS velocity showed mixed influence towards the membrane flux. Figure 6b shows the decrease in membrane flux unify either membrane flux or membrane flux at 20.0 bar osmotic pressure difference while an increase in flux at 25.0 bar. Similarly, Figure 6i shows the increase of membrane flux with the increase in DS velocity at low DS temperatures while DS velocity had a negative impact on the flux at high DS temperature. Figure 6h captures the trend of membrane flux behavior for the change in DS velocity at low and high FS temperature. The membrane flux initially decreased until the DS velocity became higher than FS velocity, where the flux started to increase. This means that for the AL-DS orientation, the positive difference between DS and FS velocity favored the membrane flux. The FS temperature positive influence when compared with variating osmotic pressure difference and DS velocity in Figure 6c,h, respectively. Figure 6f shows the positive impact on the membrane flux with the increase in FS temperature at 11 cm/s FS velocity and a slight decrease in flux at 29 cm/s FS velocity. The impact of variation for both FS and DS temperature is given in Figure 6j. The two possible scenarios to obtain high membrane flux is either high DS temperature and low FS temperature or low DS temperature and high FS temperature. High DS temperature favors the osmotic pressure difference, as seen from the van’t Hoff equation (Equation (2)) and therefore resulting in a higher membrane flux at 20 °C FS temperature. However, a high FS temperature also had a positive influence on the membrane flux. This can be explained with the thermal-osmosis phenomena in which water moves from the warmer side to the cooler side [44]. Since the water permeates from the feed side to the draw side, the increase in FS temperature at 20 °C DS temperature results in an increase in the membrane flux.

The results from the RSM study identified the optimum operating conditions for maximizing the membrane flux. Moreover, it enabled the study of the impact of each input parameter on the response (flux). The impact of the input variables on the membrane flux was in agreement with the sensitivity analysis conducted on the ANN model using its weights, as Figure 6 showed the highest impact on the flux resulted from the osmotic pressure difference, FS velocity, and DS velocity. Furthermore, a sensitivity analysis was only able to provide the relative importance of the variable, whereas RSM also identified whether the variable had a positive or negative influence on the membrane flux.

## 4. Conclusions

In this study, a combined ANN-RSM approach was used to model and optimize the membrane flux for the forward osmosis process. First, the ANN model was built using a published study in which the velocity and temperature of both FS and DS were investigated simultaneously. RSM model was built with the help of the Box-Behnken design in which responses were evaluated using the ANN model. Some of the conclusions drawn from this study are as follows:

- A high-performance ANN model was established using the published study with an overall R^2^ of 0.98036, which was validated and tested with the help of the experimental data.
- The weights of the ANN model were analyzed to investigate the sensitivity analysis of the model. The osmotic pressure difference, FS velocity, and DS velocity were found to be the highest and almost equally important operating conditions, which has an effect on the membrane flux.
- The RSM model (R^2^ = 0.9408) was used to optimize and further study the impact of the variables in terms of positive or negative influence on the membrane flux. The increase in osmotic pressure difference and FS velocity were always found to have a positive impact on the membrane flux, while the other variables had a mixed influence. The highest membrane flux (55 LMH) based on the response surface plots was obtained for the case of 25 bar osmotic pressure difference, 29 cm/s DS velocity, 20 cm/s FS velocity, 26 °C of both FS and DS temperature.

The study showed that ANN is a powerful tool to model with incomplete or missing data. Moreover, it can be combined with popular techniques such as RSM to predict and optimize the response over a range of operating conditions. In the future, the study can be further improved by the addition of specific energy consumption and reverse salt flux, which will help further optimize the process by minimizing the two variables.

## Figures and Tables

**Table 1:** Summary of the artificial neural network (ANN) model developed for the membrane processes.

Process	Input	Output	Network Architecture	Activation	Training Algorithm	Performance	References
Ultrafiltration (UF) of bleach plant effluent	pressure, tube flow velocity, the concentration ratio of the effluent	rejection of chemical oxygen demand (COD), membrane flux	3-8-2	log-sigmoid	Levenberg-Marquardt	Relative deviation = 12%	[8]
Pilot and full-scale filtration of municipal drinking water	influent flow rate, feedwater flow rate, membrane flux, operation time, pH, total dissolved solids (TDS), UV254, temperature	membrane resistance	8-8-1	log-sigmoid	Levenberg–Marquardt	Relative error = 5%	[11]
Reverse osmosis (RO) water desalination unit	feed pressure, temperature and salt concentration	permeate rate	3-5-1	log-sigmoid	Levenberg-Marquardt	R^2^ = 0.998	[14]
Filtration of sodium and magnesium chloride solutions	feed pressure, membrane flux, concentration	rejection	3-4-1	log-sigmoid	Bayesian Regularization	Absolute deviation < 5%	[10]
Removal of organic micropollutants by nanofiltration (NF)	membrane salt rejection, molecular length, equivalent width, hydrophobicity	rejection	4-2-1	tan-sigmoid	Levenberg–Marquardt	R^2^ = 0.97	[21]
Hybrid microfiltration (MF) to study membrane fouling	time, adsorbent type, membrane type, pore size, surfactant type, concentration	transient flux	6-6-3-1	tan-sigmoid	-	R^2^ = 0.986	[12]
RO desalination pilot plant	feed concentration, temperature, flow rate, pressure	membrane flux, rejection	4-5-3-1	log-sigmoid	Levenberg-Marquardt	R^2^ = 1	[15]
micellar-enhanced UF of synthetic wastewater containing lead ions	pH, feed concentration, surfactant to metal molar ratio	membrane flux, rejection rate	3-5-2	log-sigmoid	Levenberg-Marquardt	R^2^ = 0.9254 R^2^ = 0.9813	[13]
Separation of particulate suspensions using MF with turbulence promote	Inlet velocity, transmembrane pressure (TMP), concentration	flux improvement efficiency	3-12-1	log-sigmoid	Gradient descent	R^2^ = 0.9891	[17]
Pilot plant filtration of polyethylene glycol (PEG)	TMP, crossflow velocity (CFV), time	membrane flux	3-5-1	tan-sigmoid	Levenberg-Marquardt	R^2^ = 0.9977	[9]
FO desalination of groundwater	feed CFV and temperature, draw solution CFV and temperature	reverse solute flux selectivity (RSFS)	4-8-1 4-7-1	exponential	BFGS quasi-Newton backpropagation	R^2^ = 0.9943 R^2^ = 0.9988	[19]
Small scale pilot plant seawater desalination plant	power, temperature, conductivity	Pressure, flow	3-71-171 3-69-13-1	sigmoid	Resilient backpropagation algorithm	Mean absolute error = 0.405 % Mean absolute error = 0.867 %	[18]
Vacuum membrane distillation	feed inlet temperature, feed flow rate, membrane length	membrane flux, specific thermal energy consumption	3-7-2	tan-sigmoid	Levenberg-Marquardt	R^2^ = 0.9936 R^2^ = 0.9645	[16]
Modeling of Lab-scale forward osmosis desalination	membrane type, membrane orientation, feed molarity, draw molarity, molecular weight, feed velocity, draw velocity, feed temperature, draw temperature	membrane flux	9-25-25-40-1	log-sigmoid, tan-sigmoid, log-sigmoid	Levenberg-Marquardt	R^2^ = 0.973	[20]

**Table 2:** Variables for the artificial neural network (ANN) model and their ranges.

Type	Variables	Symbol	Range	Unit
Input	Osmotic pressure difference	Δπ	20.00–25.37	bar
	Feed solution (FS) velocity	$v_{FS}$	11.05–29.45	cm/s
	Draw solution (DS) velocity	$v_{DS}$	11.05–29.45	cm/s
	FS temperature	$T_{FS}$	20–32	°C
	DS temperature	$T_{DS}$	20–32	°C
Output	Membrane flux	$J_{w}$	10.0–48.0	LMH

**Table 3:** Overall and individual phase performances of the optimum artificial neural network model.

Performance	Dataset			
	Training	Validation	Test	All
Mean squared error (MSE)	0.13268	1.13577	0.32092	0.24241
Root mean squared error (RMSE)	0.36426	0.60354	0.56649	0.49235
Sum of squared error (SSE)	8.22636	7.95036	2.24641	18.42314
R-value	0.99953	0.99647	0.99888	0.99013
R^2^	0.99906	0.99295	0.99776	0.98036
Adjusted R^2^	0.99898	0.95771	0.98657	0.97895

**Table 4:** Optimal values of weights and biases obtained in the training phase.

Input weight Matrix, IW	IW{1,1} =				
**{Destination: Hidden layer**	3.4157	−0.5880	0.9954	1.1900	−1.4431
**Source: Inputs}**	2.7786	−3.3672	−1.0787	−1.5737	0.0933
	−1.1643	−2.6092	2.8253	−0.3323	1.6905
	3.1871	3.5976	2.7567	−1.8359	0.1077
	0.1369	0.3260	4.0058	−1.1023	−2.7599
	−3.7725	−0.1537	0.1178	−0.1366	−0.5589
	−3.3965	−0.5918	2.1567	4.4484	2.0689
	−1.0245	4.2560	−0.8856	0.8582	−1.1337
	0.0484	1.1973	−1.7707	0.2235	−0.2009
	0.4607	2.5649	−0.4886	0.0249	1.1392
**Bias vector, b**	b{1} =				
**{Destination: Hidden layer}**	−1.2832				
	−1.4174				
	1.4586				
	−1.2827				
	−2.6367				
	1.5104				
	−1.2738				
	0.1608				
	−0.5107				
	2.9615				
**Layer weight matrix, LW**	LW{2,1} ^T^				
**{Destination: Output layer**	0.5896				
**Source: Hidden layer}**	−0.2121				
	0.5402				
	0.1981				
	0.0384				
	−0.3912				
	−0.0861				
	−0.1507				
	0.7699				
	0.2288				
**Bias scalar, b**	b{1} = − 0.0714				
**{Destination: Output layer}**					

^T^ transpose of the matrix.

**Table 5:** Box-Behnken design and response (membrane flux) from the optimum artificial neural network model.

StdOrder	Factors					Response
	Osmotic Pressure Difference (bar)	Feed Velocity (cm/s)	Draw Velocity (cm/s)	Feed Temperature (°C)	Draw Temperature (°C)	Membrane Flux (LMH)
1	20.0	11	20	26	26	4.7
2	25.0	11	20	26	26	41.0
3	20.0	29	20	26	26	18.1
4	25.0	29	20	26	26	52.8
5	22.5	20	11	20	26	11.1
6	22.5	20	29	20	26	21.8
7	22.5	20	11	32	26	14.5
8	22.5	20	29	32	26	26.8
9	22.5	11	20	26	20	16.1
10	22.5	29	20	26	20	24.9
11	22.5	11	20	26	32	3.4
12	22.5	29	20	26	32	30.8
13	20.0	20	11	26	26	20.8
14	25.0	20	11	26	26	41.6
15	20.0	20	29	26	26	7.8
16	25.0	20	29	26	26	54.4
17	22.5	20	20	20	20	10.9
18	22.5	20	20	32	20	23.2
19	22.5	20	20	20	32	17.8
20	22.5	20	20	32	32	17.5
21	22.5	11	11	26	26	18.6
22	22.5	29	11	26	26	17.7
23	22.5	11	29	26	26	11.4
24	22.5	29	29	26	26	19.0
25	20.0	20	20	20	26	14.5
26	25.0	20	20	20	26	45.1
27	20.0	20	20	32	26	14.0
28	25.0	20	20	32	26	46.8
29	22.5	20	11	26	20	15.0
30	22.5	20	29	26	20	35.5
31	22.5	20	11	26	32	29.1
32	22.5	20	29	26	32	18.0
33	20.0	20	20	26	20	12.8
34	25.0	20	20	26	20	37.8
35	20.0	20	20	26	32	12.4
36	25.0	20	20	26	32	46.7
37	22.5	11	20	20	26	3.3
38	22.5	29	20	20	26	19.8
39	22.5	11	20	32	26	17.7
40	22.5	29	20	32	26	17.4
41	22.5	20	20	26	26	17.6

**Table 6:** Analysis of variance (ANOVA) table for the response surface modeling.

Source	DF ^a^	SS ^b^	MS ^c^	F-Value	*p*-Value	R^2^	Adj. R^2^
Model	20	6919.03	345.95	19.85	*p* < 0.001	94.08%	89.34%
Residual	25	435.72	17.43				
Total	45	7354.74					

^a^ Degree of freedom. ^b^ Sum of squares. ^c^ Mean squares.

## Data Availability

The data presented in this study are available as Supplementary Material.

## Supplementary Materials

The following are available online at https://www.mdpi.com/2077-0375/11/1/70/s1, Excel sheet 1: Training_data, Excel sheet 2: Validation_data, Excel sheet 2: Testing_data.
